# Light environment affects the efficiency of surgical suture training

**DOI:** 10.1186/s12909-024-05407-0

**Published:** 2024-04-16

**Authors:** Yuan Gu, Lihua Xie, Xianzhe Huang, Chan Liu, Zhengxiao Ouyang, Liqin Yuan, Wenzhao Li

**Affiliations:** 1 Department of Orthopedics, The Second Xiangya Hospital, Central South University, 139 Renmin Road, Changsha, 410011 Hunan P.R. China; 2 Department of Orthopedics, Nanfang Hospital of Southern Medical University, Guangzhou, Guangdong P.R. China; 3 Blood Purification Center, The Second Xiangya Hospital, Central South University, Changsha, 410011 Hunan P.R. China; 4 Department of International Medical, The Second Xiangya Hospital, Central South University, Changsha, 410011 Hunan People’s Republic of China; 5 Department of General Surgery, The Second Xiangya Hospital, Central South University, 139 Renmin Road, Changsha, 410011 Hunan P.R. China

**Keywords:** Illumination environment, Illuminance, Color temperature, Sentiment, Surgical suturing training

## Abstract

**Background:**

Suture knotting is the basis of surgical skills. In the process of surgical skills learning, the surrounding environment, especially the light, will affect the efficiency of learning. This study investigated the effect of optical environment on the learning of stitching and knotting skills.

**Methods:**

A total of 44 medical students were randomly divided into four groups and participated in the study of suture knotting in four different optical environments. During the process, we assess objective pressure level by testing salivary amylase activity Likert scale and objective structured clinical examination (OSCE) was used to estimate the subjective psychological state and overall skill mastery in surgical suturing respectively.

**Results:**

Under high illumination conditions (700 lx), the salivary amylase activity of the high color temperature group (6000 K) was significantly higher than that of the low color temperature group (4000 K) (*p* < 0.0001). Similarly, under low illumination (300 lx), the salivary amylase activity of the high color temperature group was also significantly higher than that of the low color temperature group (*p* < 0.05). The student under high illumination conditions (700 lx) and the low color temperature (6000 K) have an autonomy score between 37–45, which is significantly higher compared to the other three groups (*p* < 0.0001). Group 2 has an average OSCE score of 95.09, which were significantly higher than those of the other three groups (*p* < 0.05).

**Conclusion:**

High illumination combined with low color temperature is considered as the optimal training conditions, promoting trainees' optimism, reducing stress levels, and enhancing learning efficiency. These results highlight the pivotal role of light environment in improving the quality and efficiency of surgical skills training.

**Supplementary Information:**

The online version contains supplementary material available at 10.1186/s12909-024-05407-0.

## Introduction

In recent years, the advancement of surgical techniques has highlighted the importance of providing efficient and practical training in basic surgical skills to medical students and junior surgeons. Among these skills, surgical suturing is considered the most fundamental, and its mastery is essential for all aspiring medical professionals [1]. Learning the techniques in a more suitable environment is associated with the advantages of a better mastery for learners and better patient safety [2]. Indeed, the effectiveness of a learning activity and its quality, is associated with the emotional states that learners experienced [3]. In the learning of surgery skill, the trainee's emotional state or "sentiment" can influence their perception and attitudes [4]. Sentiment includes the trainee’s emotions, feelings, mood, and attitude towards the operation or practice. Therefore, emotional factors are closely linked to the effectiveness of surgical suture training, as they reflect the trainee's physiological and psychological state and can impact skill acquisition.

Vision is one of our most important sensory organs, the optical environment can stimulate or induce emotional responses in trainees during surgical suture training. This highlights the need to develop the training optical environment. The illumination system in an operating room is a crucial design factor for the training optical environment, as it not only impacts visual response but also non-visual responses such as performance, emotion, and attention.

**Fig. 1:**
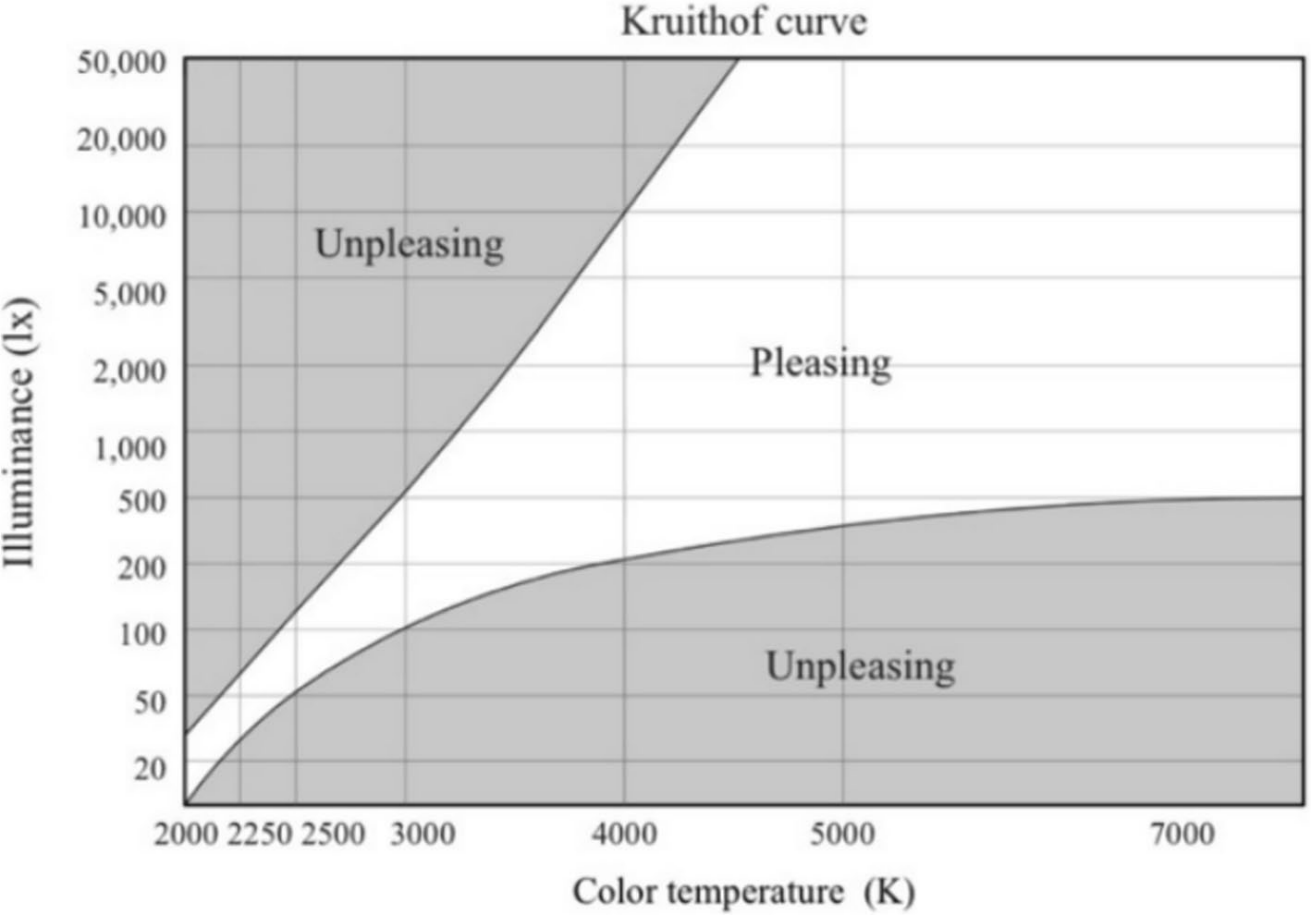
The Kruithof curve

Up to now, a number of researches have investigated the effects of the color temperature of illumination environments on mental activity. The parasympathetic and sympathetic nervous systems are both enhanced in high color temperature conditions, while drowsiness is more likely happen under lower temperature lighting [5–7]. It seems that lower color temperature is more relax and higher color temperature may arouse tension. Proper illumination can enhance the room brightness, reduce stress, and increase comfort. Scholars concur that matching light intensity and color temperature is an effective method to creating a "comfort zone" within the illumination environment. The Kruithof curve indicates that high or low illuminance, combined with high or low color temperature, can create a pleasing area [8]. Figure 1 illustrates these areas defined by Kruithof.

Traditional illumination research focused on subjective evaluation experiments. However, the lack of data repetition, accuracy, and traceability in these experiments has limited the universality of the results. Therefore, objective physiological evaluation is becoming the mainstream in illumination testing. Different illumination environments will have different influences on human sympathetic nerves. Saliva has been used to evaluate both acute and chronic stresses [9]. Several salivary immune biomarkers (including salivary amylase) are considered to be sensitive and reliable markers of mental stress in adult [10]. When the sympathetic nerves excited, salivary amylase secretion will increase, salivary amylase activity will increase, and stress will increase [11]. By measuring the activity of salivary amylase by salivary amylase meter, the stress state of the subjects in different light environments can be observed, and the physiological response of the subjects can be objectively evaluated. A moderate stress status can be achieved by creating an ideal illuminating condition, thus forming a certain learning motivation and improving learning efficiency.

Different illumination environments can influence the emotional state of trainees, thereby potentially affecting their learning ability during skill training. This study investigates how illumination conditions affect the effectiveness of surgical suturing skills training by objectively and subjectively assessing physiological and psychological responses, as well as mastery of the skill after training. The primary objective of an ideal operating room illumination environment is to provide comfort. This paper aims to determine a suitable illumination environment for surgical suturing skills training based on Kruithof's pleasure zone theory, considering the impact of different illumination and color temperature combinations on comfort.

## Methods

The study assessed the surgical suturing abilities of medical students in four illumination environments, consisting of different combinations of brightness and color temperatures in various levels. The training process was evaluated through objective structured clinical examination (OSCE) test scores, salivary amylase activity measurements, and Likert scales. OSCE scores were used to assess the skill criteria of the participants. Salivary amylase activity was used to test the stress levels of the participants. And Likert scales were used to assess the subjective feelings of the participants. Additionally, the study analyzed the impact of illumination on the efficiency of surgical suturing skills training, in order to recommend the optimal illumination conditions for improving participants' optimism, skill operation state, and learning efficiency.

Given the prevalence of smartphones and WeChat in China, we used the WeChat app and its 'Solitaire Manager' applet for easy recruitment and random grouping. Before recruiting participants, we contacted the monitors of the first-year clinical students through the Academic Affairs Department of Second Xiangya Hospital. The recruitment WeChat questionnaire was sent by the monitors to the class WeChat group to ensure that all students received the recruitment information. The questionnaire included a brief description of the study, collection of baseline information (Name, Gender, Age) and the following questions 1. whether they had passed the licensing exam, 2. whether they had attended surgical suturing training in the past, 3. whether they were in their first year of clinical practice, and 4. whether they were willing to participate in the study. The eligibility criteria were "first year of clinical internship, not having passed the licensing examination, and not having participated in surgical suturing training", and a total of 44 medical students from the Department of Clinical Medicine, Second Xiangya Hospital, Central South University were enrolled in the study.

We then divided the participants into groups. First, all participants were formed into a WeChat group, and then the number of participants and groups were selected in the applet, and the system would automatically randomly divide the participants into 4 groups (11 students in each group).The study was approved by Ethics Committee of the Second Xiangya Hospital of Central South University (LYF2023127). And informed consent was obtained from all subjects.

The training place was the demonstration classroom in the clinical skills training Center of the Second Xiangya Hospital. The demonstration classroom had the same size and teaching equipment placement. It was designed as an enclosed space without natural light, and all the Windows were covered by curtains. The lighting system consists of 6 groups of luminaires at the top of the classroom, each containing 3 LED tubes (Philips TLD/36 W/830; Philips TLD/36 W/840; Philips TLD/36 W/865). Each group of LED tubes is connected to a stepless dimmable colour mixing LED driver (Chiwang, YM 18-40W*2/3), which allows the illumination and colour temperature of the LED tubes to be continuously adjusted. Meanwhile, the ambient illuminance is measured by an LMT PO1704 lux meter (0–99999 lx, ± 0.01 lx) and the colour temperature by a TES-136 colour meter (0–9999 K, ± 0.02 K). Table 1 shows the four lighting environments set up in the four demonstration classrooms for the experimental group. In this study, we set the illumination contrasts of 700 lx and 300 lx, and the color temperature contrasts of 6000 K and 4000 K. All four combinations of illumination and color temperature fell within Kruithof's pleasure zone. Meanwhile, the training content and the surgical suture instruments used were also the same for all groups. The suture materials were fresh blocks of porcine tissue including skin, subcutaneous tissue and muscle layers. The surgical instruments, including scalpels, needle holders, forceps and suture scissors, were new instruments manufactured by Xinhua Medical Instrument Company. The sutures were all 2–0 Ethicon Mousse non-absorbable sutures (Johnson & Johnson, SA845G). The training session took place from 8 am to 12 pm. The training schedule consisted of the following activities: 8 am to 9 am, explaining the basic knowledge of surgical suture and demonstration of suture operation; 9 am to 10 am, each student will practice alone. 10 am to 10:30 am, each student will answer questions and give feedback. 10:30 am to 11 am., practice session for students to implement the feedback received. From 11:00 am to 12:00 am is test time. The test sequence of each group was the same: 1. Ten minutes of quiet rest. 2. Salivary amylase activity test for 5 min; 3. A subjective questionnaire (Likert scale, Table 2) about "comfort", "mastery" and "suture skill learning" was completed within 5 min; 4. An OSCE test evaluating each student's suturing ability, lasting for 30 min. The scores were scored by an attending surgeon with more than 5 years of experience.

**Table 1:** Illumination environment of each group

Group	Illuminance/lx	Color Temperature/K
Group 1	700	6000
Group 2	700	4000
Group 3	300	6000
Group 4	300	4000

**Table 2:** subjective questionnaire (Likert scale)

Questions	Grading				
	0	1	2	3	4
Q1. I felt very focused during the suture training					
Q2. During the training, I never had sore eyes					
Q3. I felt very calm during the training					
Q4. No sense of time passing during training					
Q5. During the actual operation, I never felt nervous and discomfort such as palpitation and shortness of breath					
Q6. Before the actual operation, I felt confident that I could complete the operation well					
Q7. Feel calm even when encountering difficulties during operation					
Q8. The visual field was clear and comfortable during operation					
Q9. Be able to recall the relevant training content completely when encountering difficulties in the operation process					
Q10. After the operation, I felt very calm, without any discomfort such as palpitation, cold hands and feet					
Q11. There was no abnormal discomfort in the eyes after the operation					
Q12. After completing the operation, I am confident that I can memorize the training content completely and apply it to work					

0 strongly disagree, 1 partially disagree, 2 don’t agree or disagree, 3 partially agree, 4 strongly agree

Before entering the classroom at 8 o'clock, participants were first kept at rest for 5 min under illumination conditions (3000 K, 200 lx) to test their salivary amylase activity and evaluate their stress at rest. Then, they entered four different lighting environments to complete corresponding training content and undergo another salivary amylase activity test to assess stress under various illumination environments. Students were asked to refrain from smoking for 2 h before saliva collection. Posture during saliva collection was standardized. The body was upright with the head tilted forward to allow saliva to accumulate on the floor of the mouth. The patient then spat the saliva into a 5 ml disposable plastic container. The samples were then centrifuged at 1036 g for 15 min and stored at -80℃ until further testing. Salivary amylase activity was determined by visible spectrophotometry, and an amylase activity assay kit (Solarbio, BC610) was used in our study.

The Likert scale (Table 2) comprises 12 subjective questions on 'comfort', 'mastery', and 'suture skill learning'. Each question has five options: strongly disagree, partially disagree, don’t agree or disagree, partially agree, and strongly agree, with a score of 0–4 assigned in that order. The questions were rationalized so that higher scores indicate stronger subjective feelings about 'comfort', 'mastery', and 'learning well', while lower scores indicate the opposite.

The Objective Structured Clinical Examination (OSCE) is a crucial assessment method used to evaluate the clinical competence of medical and allied healthcare professionals in undergraduate and postgraduate examinations. A simulation-based OSCE scenario was created consisting of 3 parts for the evaluation of surgical suture skills. The OSCE scoring table can be found in appendix 1.

Part 1: Participants complete the suture of a 10 cm long linear wound on the skin model by intermittent suture, all of which are tied with a square knot.

Part 2: Participants suture the 15 cm long subcutaneous tissue on the skin model with continuous sutures and fix sutures properly.

Part 3: Participants complete three horizontal and three vertical mattress sutures and make two manual knot tying.

This OSCE test scenario is closely aligned with the training content of the participants. The test scores of each student are scored by an attending surgeon who has more than 5 years of experience.

## Results

### Baseline statistics

We first collect the age, grade and gender of each student. As we carried on the questionnaire among the student who were in their first year of clinical practice, and did not participate in the surgical suture skills training before, we show the baseline of the statistics information in Table 3. As for age and gender, there is no significantly different among 4 groups. (*p* > 0.05).

**Table 3:** Baseline statistics

	Group1	Group2	Group3	Group4
Age (mean ± S.D)	24.18 ± 0.6441	24.36 ± 0.4322	24.45 ± 0.4126	24.55 ± 0.7671
Gender (female/male)	5/6	6/5	5/6	6/5

#### Determination of salivary amylase activity

The results of salivary amylase activity for each group are presented in the Table 4, with values expressed in KU/L. This unit represents the activity of amylase in saliva, where 1 U/ML is equivalent to 1KU/L. The assessment of stress was based on the change in salivary amylase activity, which was calculated using the formula N = (A-B)/A *100%. Here, N represents the percentage change in salivary amylase activity, A is the measurement taken before exposure to the designated light condition, and B is the measurement taken after exposure. A positive change rate indicated that stress had been relieved (sympathetic quiescence) as salivary amylase activity decreased, while a negative change rate indicated increased stress (sympathetic excitation) as salivary amylase activity increased under experimental light conditions.).

**Table 4:** Changes of amylase activity in each group

Group	Quiescence	After training	*N*
Group 1	47.35	59.08	-24.78%
Group 2	41.58	32.83	21.04%
Group 3	43.56	53.16	-22.04%
Group 4	40.87	35.97	11.99%

Table 4 displays the mean values for the resting and post-training salivary amylase activity of participants, as well as their respective rates of change, observed under each of the four illumination environments. It should be noted that the maximum difference in the participants' resting salivary amylase activity was approximately 25 KU/L, which can be attributed to differences in basal stress levels. So, every group’s measured values were averaged. Analysis of the research data revealed that groups 2 and 4 experienced a reduction in stress caused by surgical suture training, with positive changes of 21.04% and 11.99% in salivary amylase activity. Conversely, group 1 and group 3 exhibited negative rates of change in salivary amylase activity, with rates of -24.8% and -17.5% respectively. This indicates an increase in stress during surgical suture training in these two illumination environments.

**Fig. 2:**
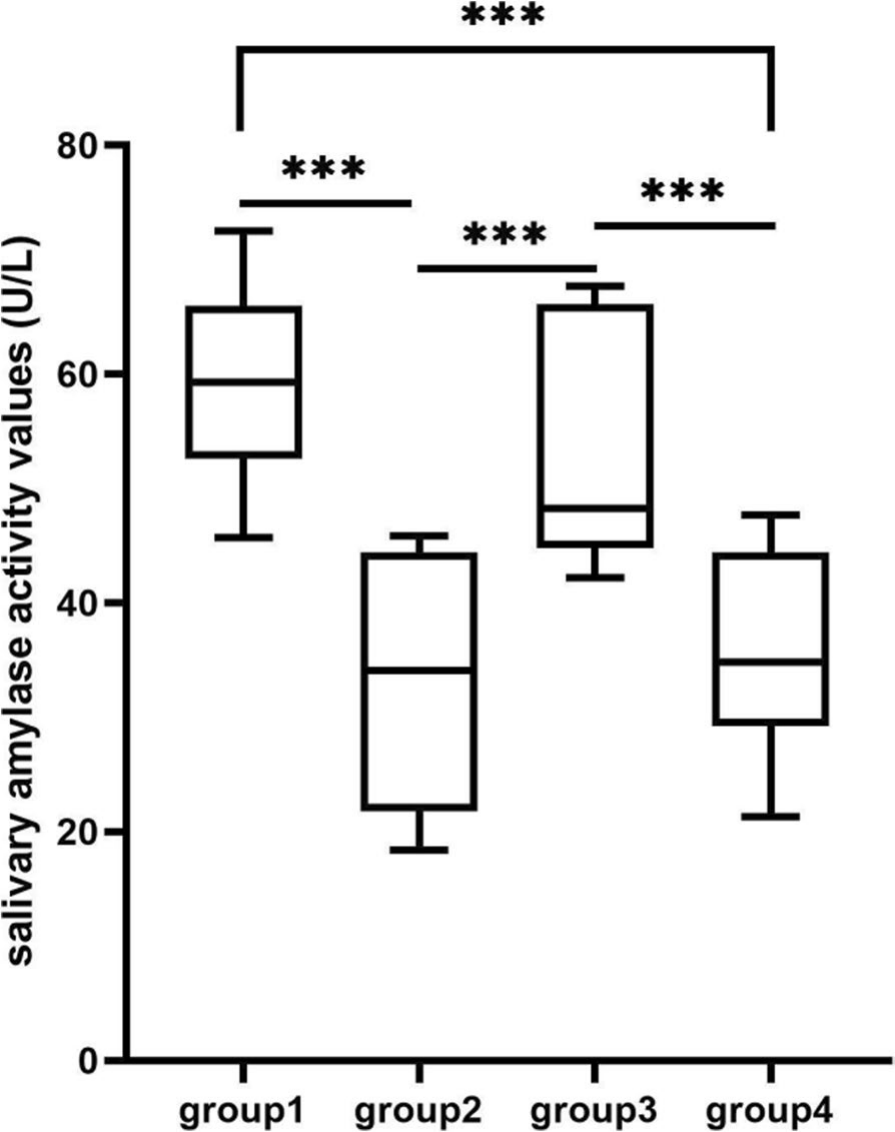
Comparison of salivary amylase activity under different illumination conditions.

The salivary amylase activity of each group was compared (Fig. 2), and T-test was performed (Table 3). In terms of color temperature, at high color temperature (6000 K), there is no significant difference between group1 at high illumination and group 3 at low illumination. Similarly, at low color temperature (4000 K), there was no significant difference between case 2 at high illumination and case 4 at low illumination. On the other hand, under the condition of high illumination (700 lx), the salivary amylase activity of group1 with high color temperature was significantly higher than that of group2 with low color temperature (t = 6.274, df = 19.71, p < 0.0001). Under low illumination (300 lx), the activity value of high color temperature case 3 was significantly higher than that of low color temperature case 4 (t = 4.205, df = 19.70, p = 0.0004 (p < 0.05)). The results showed that salivary amylase activity changed with color temperature, and light had little effect on amylase activity. In particular, the salivary amylase activity values of group1 and group3 were very high under high color temperature conditions.

#### Subjective questionnaire (Likert scale) evalution and analysis

**Fig. 3:**
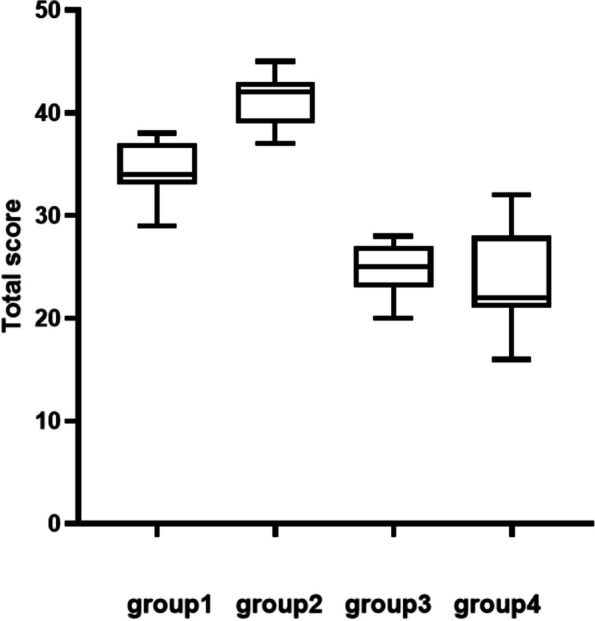
Comparison of subjective questionnaire scores under different illumination conditions

The scale had a total score of 48 points. Scores for each group were compared under four different lighting environments (Fig. 3), and t-test was conducted (Table 5).

**Table 5:** T-test of subjective questionnaire (Likert scale) scores among groups

	*p*	t	df
group1&group2	< 0.0001	6.426	19.46
group1&group3	< 0.0001	8.287	19.78
group1&group4	< 0.0001	5.98315.86	15.86
group2&group3	< 0.0001	15.84	19.92
group2&group4	< 0.0001	10.52	14.40
group3&group4	0.6385	0.4796	14.91

Under high illumination conditions (700 lx), the autonomy score of Group 1 with high color temperature was significantly lower than that of Group 2 with low color temperature (t = 6.426, df = 19.46, *p* < 0.0001). However, under low illumination (300 lx), there was no significant difference between high color temperature (Group 3) and low color temperature (Group 4) (t = 0.4796, df = 14.91, *p* = 0.6385). When considering high color temperature (6000 K), the autonomy score of Group 1 under high illumination was significantly higher than that of Group 3 under low illumination (t = 8.287, df = 19.78, *p* < 0.0001). Similarly, in the low color temperature condition (4000 K), the autonomy score of Case 2 under high illumination was significantly higher than that of Group 4 under low illumination (t = 10.52, df = 14.40, *p* < 0.0001).

**Fig. 4:**
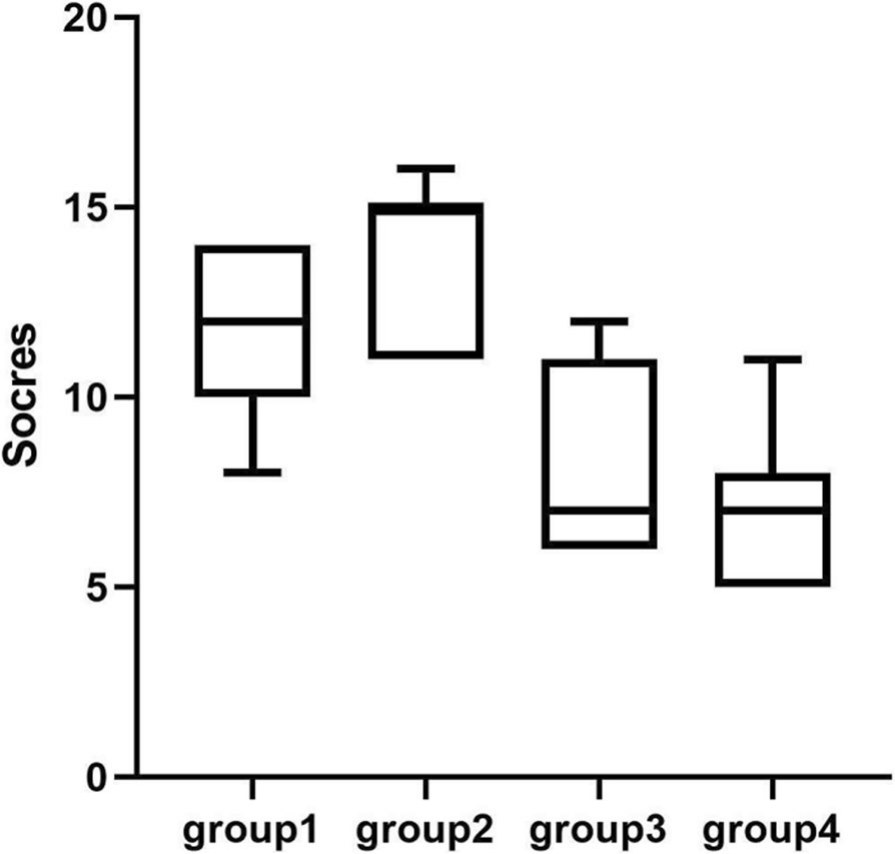
Comparison of subjective questionnaire during training

**Fig. 5:**
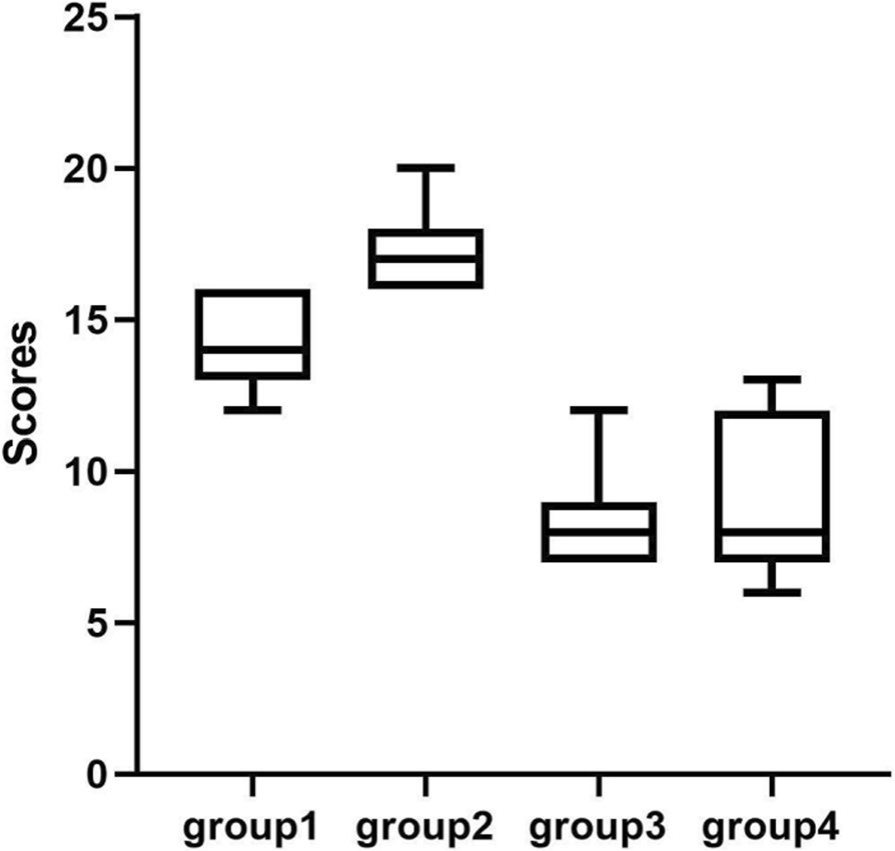
Comparison of subjective questionnaire during operation

**Fig. 6:**
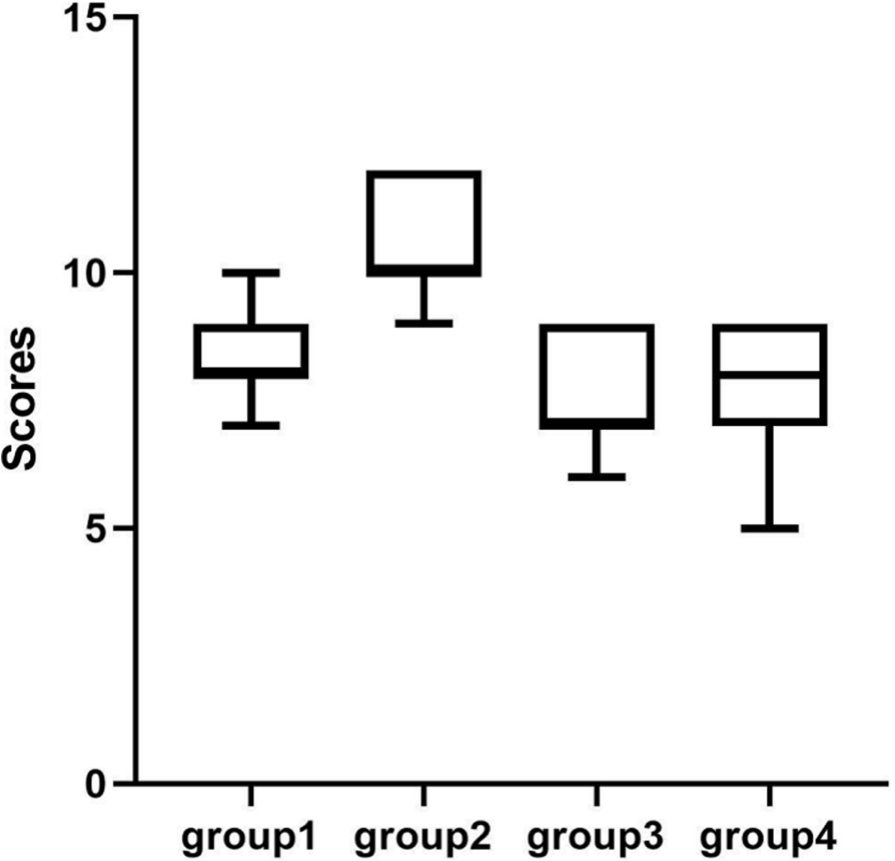
Comparison of subjective questionnaire after operation

We divided the Likert scale into 3 parts: during the training, during the operation and after the operation. In each part, we set different question to evaluate trainee's subjective felling. These questions asked about their confidence, operation situation, emotion and physiological reaction during the whole examination. We compared the scores of each part in different groups. During the training(Fig. 4 and Table 6) and operation (Fig. 5 and Table 7), students in group2 shown a significantly positive reaction compared with other groups. When the training and operation was done, there was no different among the student in group1, group2 and group3 (Fig. 6 and Table 8). After the operation, students in group2 seems had more confidence in future surgical operation.

**Table 6:** T-test of subjective questionnaire during training

	*p*	t	df
group1&group2	0.0037	2.257	19.99
group1&group3	0.0353	t = 3.290	19.61
group1&group4	< 0.0001	t = 4.970	20.00
group2&group3	< 0.0001	t = 5.410	19.48
group2&group4	< 0.0001	t = 7.284	19.99
group3&group4	0.2034	t = 1.316	19.61

**Table 7:** T-test of subjective questionnaire during operation

	*p*	t	df
group1&group2	0.0001	t = 4.834	19.96
group1&group3	< 0.0001	t = 8.589	19.83
group1&group4	< 0.0001	t = 5.758	15.97
group2&group3	< 0.0001	t = 13.36	19.62
group2&group4	< 0.0001	t = 9.195	15.53
group3&group4	0.5622	t = 0.5912	16.89

**Table 8:** T-test of subjective questionnaire after operation

	*p*	t	df
group1&group2	< 0.0001	t = 5.213	19.10
group1&group3	0.1116	t = 1.668	19.10
group1&group4	0.1291	t = 1.596	16.83
group2&group3	< 0.0001	t = 6.236	20.00
group2&group4	< 0.0001	t = 5.605	18.92
group3&group4	0.8708	t = 0.1648	18.92

These fundings indicate that high illumination can significantly improve the self-score of knotting learning, while low color temperature can improve the comfort and efficiency in learning when combined with high illumination.

## Objective structured clinical examination (OSCE) of surgical suture skills

**Fig. 7:**
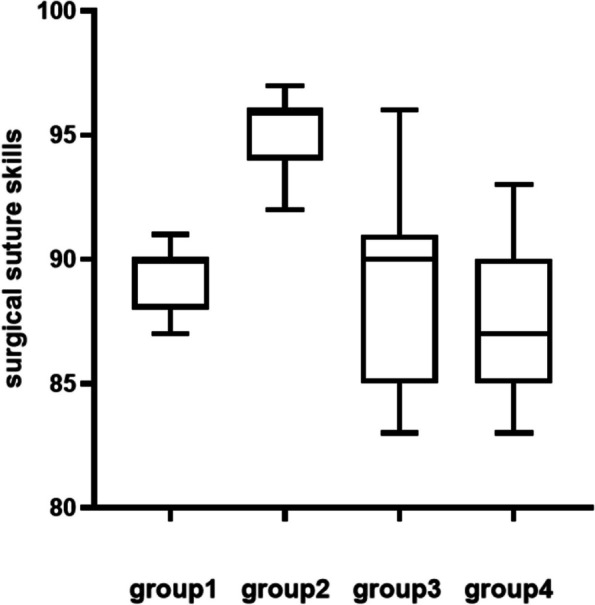
OSCE scores among groups

The specific scoring criteria can be found in Appendix 1. The total score for the assessment is 100 points. The average scores for Group 1, Group 2, Group 3, and Group 4 were 89.09, 95.09, 88.91, and 87.45, respectively. Statistical analysis of the scores from the four groups (refer to Fig. 7) indicated that there was no significant statistical difference in the scores among Group 1, Group 3, and Group 4. However, the scores of Group 2 were significantly higher than those of the other three groups (*p* < 0.05). The results demonstrate that the condition of high illumination and low color temperature is more suitable for learning the knotting operation.

## Discussion

Currently, studies have shown that the environment has a significant impact on students' performance [12]. Among all environmental factors, light is considered to play one of the most important roles [12]. A study shows that compared with the other six environmental factors, the contribution rate of light to student progress is 21%, and the proportion is the highest compared to six other environmental factors [13]. The medical technology department is biased towards practical operation, so it was better that educators can manipulate the learning environment to encourage students to adopt deep learning [14, 15]. Creating a good lighting environment is an effective way to enhance students' learning enthusiasm and optimism.

This study investigated the effects of different illumination environments on surgical suturing training by examining salivary amylase activity, participants' self-evaluation questionnaire scores, and scores from the Objective Structured Clinical Examination (OSCE) surgical suturing test. Regarding the changes in salivary amylase activity, we found that the impact of illumination on the pressure of the trainees was significantly less than that of color temperature. Low color temperature was found to be more conducive to maintaining the good mentality of the participants, reducing the pressure in the training process, and achieving better training results. The participants' subjective self-evaluation scale indicated that high illumination could significantly enhance their experience of suture training, and low color temperature proved beneficial in improving comfort and operational efficiency in surgical operation learning under high illumination conditions.

After analyzing the result of the OSCE test, we found that the combination of high illumination and low color temperature resulted in higher test scores for the trainees. This suggests that participants mater the skill better in this condition. Overall, the current findings indicate that high illumination and low color temperature lighting conditions within the Kruith of pleasure zone are the most ideal illumination conditions for surgical suture training.

However, the universality of the study's findings is still affected by some factors. Firstly, the experiment was limited to a group of surgical trainees in Changsha, Hunan Province, China. Although the sample size is sufficient to study the effect of illumination environment, it is still not enough to promote the research results in a large number of medical students in China. In the future, more experiments need to be carried out in more training participants and the results need to be further analyzed. Secondly, the ideal lighting environment may vary by culture or geographic location, as people in different cultures and regions of the world will have different emotional responses to the same color temperature and illumination. For surgical trainees of different cultures and regions, whether the ideal training effect can be achieved under the illumination environment found in this paper needs further investigation and verification. Thirdly, the impact of this study on the classroom lighting environment is limited because this study is still considered exploratory. In future work, the color rendering index, illuminance ratio uniformity and glare can be considered to determine the ideal training lighting environment.

## Conclusions

Regarding the changes in salivary amylase activity, we found that the impact of illumination on the pressure of the trainees was significantly less than that of color temperature. Low color temperature was found to be more conducive to maintaining the good mentality of the participants, reducing the pressure in the training process, and achieving better training results. The participants' subjective self-evaluation scale indicated that high illumination could significantly enhance their experience of suture training, and low color temperature proved beneficial in improving comfort and operational efficiency in surgical operation learning under high illumination conditions. After analyzing the result of the OSCE test, we found that the combination of high illumination and low color temperature resulted in higher test scores for the trainees. This suggests that participants mater the skill better in this condition.

Overall, the current findings indicate that high illumination and low color temperature lighting conditions within the Kruith of pleasure zone are the most ideal illumination conditions for surgical suture training.

### Supplementary Information

**Supplementary Material 1.**

## Data Availability

The datasets used and analyzed during the current study are available from the corresponding author on reasonable request.

## References

[1] Dasci S, Schrem H, Oldhafer F, Beetz O, Kleine-Döpke D, Vondran F, Beneke J, Sarisin A, Ramackers W (2023). Learning surgical knot tying and suturing technique - effects of different forms of training in a controlled randomized trial with dental students. GMS J Med Educ..

[2] Shaharan S, Neary P (2014). Evaluation of surgical training in the era of simulation. World J Gastrointest Endosc.

[3] Cau M, Sandoval J, Arguel A, Breque C, Huet N, Cau J, Laribi MA (2022). Toward Optimal Learning of the Gesture in Laparoscopic Surgery: Methodology and Performance. J Clin Med.

[4] Wijesinghe K, Lunuwila S, Gamage H, Gooneratne T, Munasinghe BNL, Harikrishanth S, Nandasena M, Perera N, Jayarajah U (2023). Medical students' perception and attitudes on operating theatre learning experience in Sri Lanka. Surg Open Sci.

[5] Noguchi H, Sakaguchi T (1999). Effect of illuminance and color temperature on lowering of physiological activity. Appl Human Sci.

[6] Mukae H, Sato M (1992). The effect of color temperature of lighting sources on the autonomic nervous functions. Ann Physiol Anthropol.

[7] Deguchi T, Sato M (1992). The effect of color temperature of lighting sources on mental activity level. Ann Physiol Anthropol.

[8] Davis RG, Ginthner DN (1990). Correlated Color Temperature, Illuminance Level, and the Kruithof Curve. J Illum Eng Soc.

[9] Łoś K, Waszkiewicz N (2021). Biological Markers in Anxiety Disorders. J Clin Med.

[10] Segerstrom SC, Miller GE (2004). Psychological stress and the human immune system: a meta-analytic study of 30 years of inquiry. Psychol Bull.

[11] Behringer V, Deschner T, Möstl E, Selzer D, Hohmann G (2012). Stress affects salivary alpha-Amylase activity in bonobos. Physiol Behav.

[12] Sun B, Zhang Q, Cao S (2020). Development and Implementation of a Self-Optimizable Smart Lighting System Based on Learning Context in Classroom. Int J Environ Res Public Health.

[13] Barrett P, Davies F, Zhang Y, Barrett L (2015). The impact of classroom design on pupils’ learning: Final results of a holistic, multi-level analysis. Build Environ.

[14] Ngwira FF, Kamwaza M, Rashid S, Boby G, Kadzakumanja G (2019). Medical and allied health students’ self-regulated learning: the interplay between motivational beliefs and learning strategies. J Contemp Med Edu..

[15] Chen DP, Chang SW, Burgess A, Tang B, Tsao KC, Shen CR, Chang PY (2023). Exploration of the external and internal factors that affected learning effectiveness for the students: a questionnaire survey. BMC Med Educ.

