# Supplementary material for: Light environment affects the efficiency of surgical suture training
Source: BMC Med Educ. 2024 Apr 16;24:415. doi: 10.1186/s12909-024-05407-0 (PMC11020322; doi:10.1186/s12909-024-05407-0)
Supplement: Supplementary file 1 — Supplementary Material 1. [file 12909_2024_5407_MOESM1_ESM.pdf]

表 2-1-5　缝针缝线选择及缝合打结评分标准

项目（分）	具体内容及评分细则	满分（分）	得分（分）	备注
戴手套（10）	用手自手套袋内捏住手套套口翻折部，将手套取出	2		
	正确带好手套	5		
	手套戴好后应扎住袖口	3		
穿针（10）	持针器夹针后 1/3 处	3		
	穿针时针尖朝外	3		
	持针器夹好针后再穿线	2		
	穿针后留短线长短合适	2		
缝针选择（15）	三角针	5		
	三角针	5		
	三角针	5		
	圆针	5		
	圆针	5		
缝线选择（15）	1 号线（3–0）	5		
	1 号线（3–0）	5		
	7 号线（1–0）或 10 号线（1）	5		
	1 号线（3–0）	5		
	1 号线（3–0）	5		
缝合方式（15）	单纯间断缝合	5		
	间断垂直褥式外翻缝合	5		
	间断减张缝合	5		
	间断垂直褥式内翻缝合	5		
	单纯间断缝合	5		
打结（20）	方结或三重结	5		
	方结或三重结	5		
	三重结	5		
	方结	5		
	方结	5		
	结扎牢靠	5		
边距、针距和线头长度（15）	针距 1.0 ~ 1.2 cm，边距 0.5 ~ 0.6 cm，线头 0.5 ~ 0.8 cm	5		
	针距 1.0 ~ 1.2 cm，线头 0.5 ~ 0.8 cm	5		
	针距及边距长，缝线套橡皮管，线头 0.5 ~ 0.8 cm	5		
	针距边距无要求，线头 0.3 ~ 0.5 cm	5		
	针距边距无要求，线头尽量留短，贴近线结最好	5		
总分		100		
裁判签名				

湘雅临床技能培训教程
